# Supplementary material for: Integrated Transcriptomic and Proteomic Analyses Reveal Molecular Mechanism of Response to Heat Shock in Morchella sextelata
Source: J Fungi (Basel). 2025 Jan 18;11(1):76. doi: 10.3390/jof11010076 (PMC11766532; doi:10.3390/jof11010076)
Supplement: Supplementary file 1 [file jof-11-00076-s001.zip › Supplementary file 2.pdf]

Cultivation of Morel (*Morchella sextelata*): Key Factors

1. Humidity:

The ideal relative humidity is around 80%-90%. This helps maintain the moisture in the soil and supports the growth of the mycelium. During the fruiting stage, regular misting is necessary to ensure the environment stays moist but not waterlogged.

2. Temperature:

Mycelium stage: The ideal range is between 5°C and 20°C.

Fruiting stage: usually around 10°C to 15°C.

3. Cultivation Cycle:

Usually 4 to 6 months, depending on the growing conditions and temperature.

4. Soil Type:

Morels thrive in well-drained, loamy soils rich in organic matter. pH (6.5 - 7.5) is preferred. The soil should be loose and friable to ensure adequate airflow and moisture retention.

5. Management Process:

- Soil Preparation: Start by preparing a bed with a mix of loamy soil and organic material. The soil should be moist but not too wet.
- Inoculation: Spread the morel mushroom spawn evenly across the prepared bed or soil mixture with 200 kg per 666 m^2.
- Exogenous nutrition aiding: After 10-15 days, uniformly and orderly place the exogenous nutrient bags across the entire surface, with a quantity of 2,000 kg per 666 m^2.
- Moisture Management: Keep the soil consistently moist, particularly during the primordium and fruiting periods. Mist the soil as needed to maintain high humidity without waterlogging.
- Temperature Control: During the primordium and fruiting periods, maintain a consistent warm temperature.
- Pest and Disease Control: Monitor for pests, especially slugs and mites, and ensure that the humidity level doesn't promote mold growth. Regular inspection is crucial to prevent contamination.
- Harvesting: Morels should be harvested when fully mature but before they start to dry out. They typically reach maturity within a few weeks of fruiting.
